# Evaluation of Different Reference Based Annotation Strategies Using RNA-Seq – A Case Study in *Drososphila pseudoobscura*

**DOI:** 10.1371/journal.pone.0046415

**Published:** 2012-10-03

**Authors:** Nicola Palmieri, Viola Nolte, Anton Suvorov, Carolin Kosiol, Christian Schlötterer

**Affiliations:** Institut für Populationsgenetik, Vetmeduni Vienna, Vienna, Austria; Wayne State University, United States of America

## Abstract

RNA-Seq is a powerful tool for the annotation of genomes, in particular for the identification of isoforms and UTRs. Nevertheless, several software tools exist and no standard strategy to obtain a reliable annotation is yet established. We tested different combinations of the most commonly used reference-based alignment tools (TopHat, GSNAP) in combination with two frequently used reference-based assemblers (Cufflinks, Scripture) and evaluated the potential of RNA-Seq to improve the annotation of *Drosophila pseudoobscura*. While GSNAP maps a higher proportion of reads, TopHat resulted in a more accurate annotation when used in combination with Cufflinks. Scripture had the lowest sensitivity. Interestingly, after subsampling to the same coverage for GSNAP and TopHat, we find that both mappers have similar performance, implying that the advantage of TopHat is mainly an artifact of the lower coverage. Overall, we observed a low concordance among the different approaches tested both at junction and isoform levels. Using data from both sexes of two adult strains of *D. pseudoobscura* we detected alternative splicing for about 30% of the FlyBase multiple-exon genes. Moreover, we extended the boundaries for 6523 genes (about 40%). We annotated 669 new genes, 45% of them with splicing evidence. Most of the new genes are located on unassembled contigs, reflecting their incomplete annotation. Finally, we identified 99 additional new genes that are not represented in the current genome contigs of *D. pseudoobscura*, probably due to location in genomic regions that are difficult to assemble (e.g. heterochromatic regions).

## Introduction

RNA-Seq technology is a powerful tool for the annotation of genomes due to its potential to identify precise exon boundaries and the ability to detect lowly expressed transcripts (e.g. [1], [2], [3], [4], [5], [6]). Annotation via RNA-Seq can be performed using a reference-based approach, *de novo*, or a combined strategy [7]. The choice of strategy mainly depends on the availability of a reference genome. In a reference-based approach reads are aligned to a genomic reference using a mapper specifically designed for RNA-Seq data, followed by transcriptome reconstruction from the mapped reads. In the *de novo* approach transcripts are directly reconstructed from the reads. The major challenge in both approaches is the disentangling of different isoforms. Since the introduction of RNA-Seq many mapping tools have been developed, with TopHat [8] being among the most popular ones. For transcriptome reconstruction the most commonly used software tools are Cufflinks [9] and Scripture [10], which reconstruct a set of transcripts using reads mapped with TopHat. Although other mappers, such as GSNAP [11], have been described to be more accurate than TopHat [12], to our knowledge they have never been used in combination with the transcriptome reconstruction tools mentioned above.

Here we use RNA-Seq to improve the annotation of *D. pseudoobscura*, a frequently studied *Drosophila* species that is widely used to address questions such as the evolution of inversions (e.g. [13]), speciation (e.g. [14]) and sex chromosome evolution (e.g. [15]). The current annotation of *D. pseudoobscura* has remained almost unchanged since the first release of the genome in 2005 [16] and suffers from some important limitations: only about 25% of the genes are supported by ESTs, alternative splicing is detected for only 2% of the genes and only few genes have annotated UTRs (2%). Here we improved the annotation *of D. pseudoobscura* by exploiting the resolution power of RNA-Seq. Our study extends the gene boundaries for 40% of the genes, detects 669 new genes and reveals alternative splicing for about 30% of the multiple-exon genes. Moreover, we provide evidence for 99 additional genes located in unassembled genomic regions.

## Materials and Methods

### Sample Preparation and RNA-Seq

The *D. pseudoobscura* strains ps94 (stock number 14011-0121.94) and ps88 (stock number 14011-0121.88) were obtained from the UC San Diego *Drosophila* Stock Center. Flies were reared on standard cornmeal-molasse-yeast-agar medium and maintained at 19°C under constant dark conditions. For each strain, virgin females and virgin males were collected from 15–20 replicate vials, pooled and allowed to age for three to seven days before shock-freezing in liquid nitrogen. For extraction of RNA, females and males of each strain were homogenized in TRIzol Reagent (Invitrogen, Carlsbad, CA, USA) using an Ultra-turrax T10 (IKA-Werke, Staufen, Germany). Total RNA was extracted with TRIzol Reagent following the manufacturer’s instruction, quality-checked on agarose gels and quantified using the Qubit RNA Assay Kit (Invitrogen, Carlsbad, CA, USA). For every sex-genotype combination two replicate RNA extractions were performed, which were pooled before sequencing. Paired-end Illumina mRNA libraries were generated from 10 µg total RNA using the mRNA Sample Prep Kit (Illumina, San Diego, CA). Library construction followed the protocol of the mRNA-Seq Sample Prep Manual (Revision A) except that we used a larger insert size of 300–350 bp. Cluster amplification was performed using the TruSeq PE Cluster Kit v5 on a cluster station, and each sample was sequenced on a separate GAIIx lane using TruSeq SBS 36 Cycle Kits v5 (Illumina, San Diego, CA) (read length 101bp).

### Reference-based Annotation

**Figure 1:**
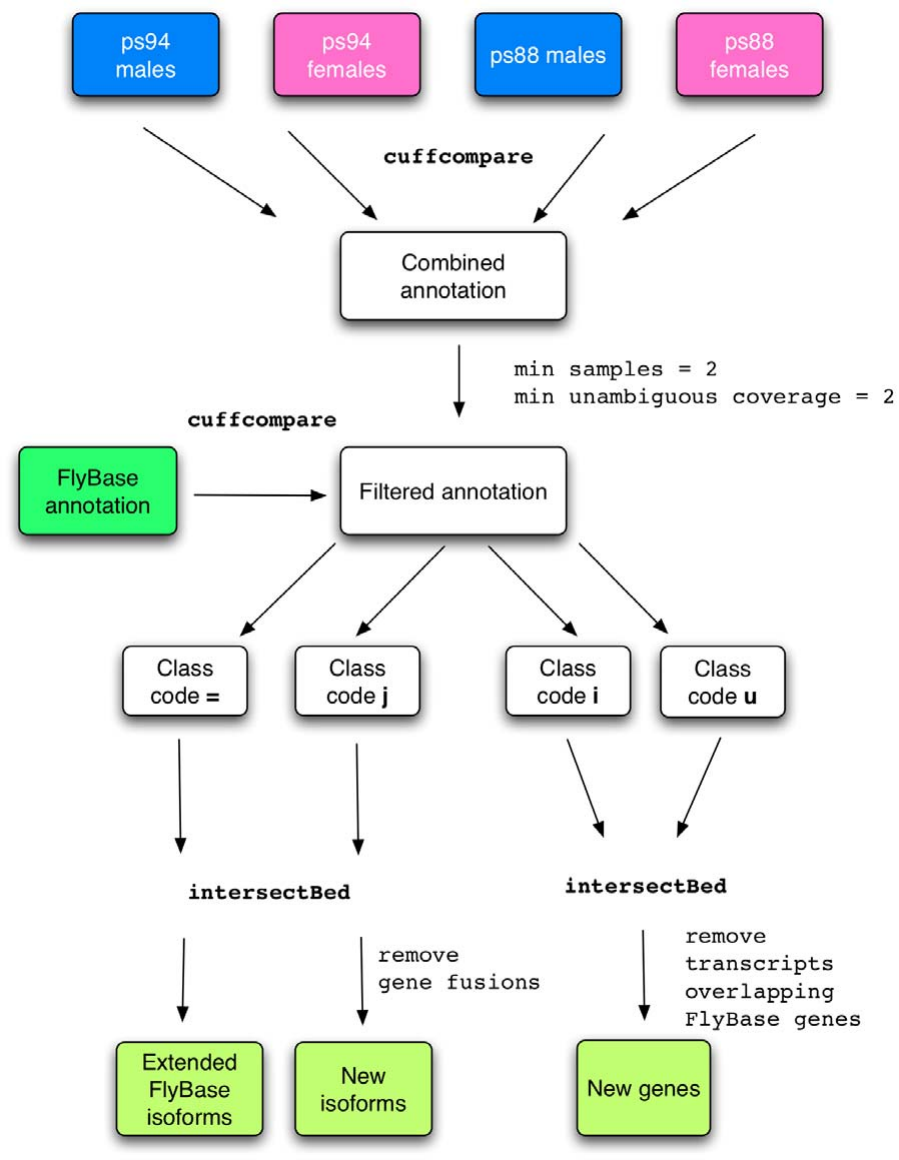
Annotation pipeline. Four samples of *D. pseudoobscura* are independently annotated with Cufflinks and then merged with cuffcompare. Isoforms confirmed in at least two samples and having at least 2-fold coverage by unambiguous reads are retained. The filtered annotation is compared against the FlyBase annotation using cuffcompare retaining extended FlyBase isoforms ( = ), new isoforms (j), new genes located in introns (i) or intergenic regions (u). Gene fusions are filtered out from the extended and new isoforms with intersectBed. New genes overlapping FlyBase genes directly or indirectly through transcripts detected in only one sample are removed using intersectBed.

Reads were trimmed using the Mott algorithm implemented in [17] (minimum read length = 40, quality threshold = 20). We used TopHat version 1.3.3 [8] (–phred64-quals) and GSNAP version 2011-11-29 [11] (–quality-protocol = illumina -A sam –N1) for mapping the reads against the *D. pseudoobscura* genome release 2.23. Cufflinks (version 1.2.1) and Scripture (version from 22-06-2010) were used for transcriptome assembly. Transcripts assembled by Scripture were clustered into genes using Genetools (http://www.broadinstitute.org/~mgarber/) (-minOverlap 0.1). We generated various annotations from the sample ps94 males using different combinations of a mapping and an assembly tool (TopHat-Cufflinks, GSNAP-Cufflinks, TopHat-Scripture), except GSNAP-Scripture, due to incompatibilities between the two programs. To validate the annotations generated in this way we used two conservative annotation-sets as a reference. The first one is based on the assumption that the *D. melanogaster* gene models are the most complete and conserved between *D. melanogaster* and *D. pseudoobscura*. Following this idea, we extracted all the ortholog gene names between *D. melanogaster* and *D. pseudoobscura* from the orthology assignments from FlyBase, and we retained only one-to-one orthologs. Using Exonerate [18] version 2.2.0 (–minintron 50 -Q protein -T dna -m protein2genome –showtargetgff –bestn 1–forwardcoordinates 0) we aligned for each ortholog-pair all the *D. melanogaster* proteins against the corresponding *D. pseudoobscura* gene region including 2 kb flanking regions. Only annotated transcripts corresponding to complete ORFs in *D. melanogaster* were retained for further analyses. A second annotation was generated using all the *D. pseudoobscura* ESTs downloaded from the NCBI database (on the 2-12-2011). We aligned the ESTs with Exonerate against the *D. pseudoobscura* genome using a stringent cutoff of 95% length. We validated the annotations generated with RNA-Seq by measuring sensitivity at base and junction levels against the orthology and EST annotation. After choosing the best mapper-assembler combination (TopHat-Cufflinks), we applied this procedure to the other three samples independently (TopHat parameters: –phred64-quals, Cufflinks parameters: –F 0). Then we combined all the annotations as depicted in Figure 1. First, we merged the four sample annotations with cuffcompare. In order to minimize annotation artifacts we selected only transcripts confirmed in at least two samples and having a coverage of uniquely mapped reads greater than two. We compared the obtained annotation with the *D. pseudoobscura* annotation release 2.23 using cuffcompare. We extracted extended FlyBase isoforms (cuffcompare category “ = ”), new isoforms (cuffcompare category “j”) of FlyBase genes, new genes located in FlyBase introns (cuffcompare category “i”) or intergenic regions (cuffcompare category “u”). Then we combined intronic and intergenic transcripts to obtain a unique set of new genes. Transcripts spanning at least two FlyBase genes in the same orientation were discarded from the sets of extended and new isoforms. Isoforms overlapping FlyBase transcripts directly or indirectly via transcripts detected only in one sample were removed from the new genes set using the intersectBed program from BEDTools [19]. We validated new genes by searching for orthologs in *D. melanogaster* using BLASTX [20] (e-value <10^−10^) and calculating their coding propensity using the Coding Potential Calculator (CPC) [21]. For newly discovered isoforms of FlyBase genes and new genes we extracted the corresponding transcript sequences and predicted ORFs using the online tool ORFPredictor (http://proteomics.ysu.edu/tools/OrfPredictor.html). Transcript-based coordinates of newly predicted ORFs were converted to genome-based coordinates and only complete ORFs were retained. Introns coordinates were derived from the exon coordinates, while UTRs were annotated by the interval difference between the exonic and the CDS region of each transcript. The final annotation is stored in the standard GTF format including the following features: exon, intron, CDS, five_prime_UTR and three_prime_UTR (Dataset S3).

### GO Analyses

All GO analyses are performed on the corresponding *D. melanogaster* orthologs using the online tool Funcassociate 2.0 [22]. Visualization of GO categories was done using the online tool AmiGO [23].

### *De Novo* Assembly

The purpose of the *de novo* assembly was to find genes not located on the current genome contigs of *D. pseudoobscura* (that we termed new-extra genes). A few software packages specialized in *de novo* transcriptome assembly have been recently developed [24], [25], [26]. Among them, Trinity has been shown to reach the highest accuracy [26], [27], although it has extensive memory requirements. For this reason, we only performed *de novo* assembly of all the unmapped reads from TopHat instead of using reads from the whole sample. This procedure was repeated for each sample independently. Subsequently, contigs were aligned to the *D. pseudoobscura* genome using BLAT and unmapped contigs were retained for every sample. In order to minimize false positives, we applied a similar filtering as done for the reference-based assembly, by selecting contigs confirmed in at least two samples using blastclust (-a 20–p F –W 32). For confirmed contigs we retained the longest one from each sample. We scanned for orthologs in *D. melanogaster* and *D. persimilis* using BLASTX (e-value <10^−10^), BLASTN (e-value <10^−10^) and Exonerate (-model protein2genome) based on the CDS found from ORFPredictor. Finally, we classified new-extra genes into coding and non-coding with the CPC software [21].

### Conservation Analysis

PhastCons [28] conservation scores relative to a multiple alignment of 15 insects were downloaded from the UCSC database (http://genome.ucsc.edu/cgi-bin/hgTables?command=start). We mapped novel exons belonging to new isoforms, new genes and new-extra genes against the *D. melanogaster* genome using BLASTN (e-value <10^−10^). For each exon we extracted the coordinates of the best unambiguous hit and calculated the average PhastCons score. We compared the exon scores to the average score distribution of introns and neutrally evolving short introns [29].

## Results

### Reference-based Assembly

Reference-based assemblies are usually performed by aligning RNA sequence reads to a reference genome using an RNA-Seq specific aligner (i.e. a spliced aligner). The mapped reads are then used by specialized software packages, such as Cufflinks [9] and Scripture [10] for gene model construction. We evaluated reference-based assemblies using four samples of *D. pseudoobscura* from two-strains (ps94, ps88) and two-sexes (no replicates). In the following we refer to each sample as: ps94 males, ps94 females, ps88 males and ps88 females.

First, we evaluated two widely used spliced aligners (TopHat and GSNAP) using a single sample (ps94 males). We chose TopHat, as it is designed to be used in conjunction with the transcriptome reconstruction tools [10], and GSNAP, as it was reported to be among the most accurate mappers in a comprehensive comparison of spliced aligners [12]. While TopHat mapped only about 55% of the reads as paired-end reads, GSNAP was able to map 86% of the reads as paired-ends (Table S1). Both software tools mapped a similar fraction of reads in additional three RNA-Seq data sets, confirming that the discrepancy was not library specific (Table S1).

Given the large discrepancy of mapped paired-end reads and a previously reported higher mapping accuracy of GSNAP for RNA-Seq reads [12], we assumed that the accuracy of the transcriptome reconstruction would depend on the correctness of the mapping step. Thus, we decided to use both TopHat and GSNAP in combination with the transcriptome reconstruction tools Cufflinks and Scripture. The combination GSNAP-Scripture was not used due to compatibility issues, as Scripture fails in merging the independent alignments of the two mates when the mapped reads from GSNAP are given to the Scripture pipeline (see http://www.broadinstitute.org/software/scripture/Walkthrough_example for the detailed Scripture pipeline). Thus, we evaluated three mapper-assembler combinations (TopHat-Cufflinks, GSNAP-Cufflinks, TopHat-Scripture).

### Evaluating the Annotation Procedure

Since the *D. pseudoobscura* genome is only incompletely annotated, we decided to validate the obtained annotations based on two different data sets. The first validation set consists of isoforms conserved between *D. melanogaster* and *D. pseudoobscura*. To obtain this set, we re-annotated one-to-one orthologous isoforms between *D. pseudoobscura* and *D. melanogaster* based on stringent criteria (see Materials and Methods, orthology annotation section, for details). The orthology annotation validation set includes 3818 genes. The second validation data set is based on all *D. pseudoobscura* ESTs from NCBI (downloaded on 02-12-2011) and contains 4825 genes. The accuracy of transcriptome reconstruction can be evaluated at different levels: a straightforward approach is to calculate the proportion of correctly recovered complete isoforms (isoform-level sensitivity), as performed in [30]. Since ESTs rarely correspond to complete transcripts, isoform-level sensitivity may be not suitable to validate our annotations. Thus, we decided for each of the three mapper-assembler combinations to evaluate the completeness of the reconstruction by measuring base-level sensitivity as described in [31] and by calculating the percentage of correctly recovered junctions. We note that our evaluation procedure does not allow the identification of false positives, thus it is not possible to decide which procedure is superior for the identification of *D. pseudoobscura* specific isoforms.

We used one of the four RNA-Seq samples (ps94 males) to compare different combinations of mappers and reference based assemblers. We found that all combinations tested were of similar quality based on our evaluation criteria, but the TopHat-Cufflinks combination consistently produced the best results for base-level accuracy and confirmed junctions, independent of the validation data set used (Table 1). We note that the junction-level accuracy is not directly comparable with the one found for TopHat in a comparative study [12], since we are evaluating the junctions belonging to gene models generated by Cufflinks, rather than the set of junctions directly produced by TopHat.

**Table 1:** Comparison of reference-based approaches.

Program combination	*vs.* orthology annotation		*vs.* EST annotation	
	Base-level accuracy (%)^1^	Confirmed junctions (%)^1^	Base-level accuracy (%)^1^	Confirmed junctions (%)^1^
TopHat + Cufflinks	83.9	75.8	68.9	63.0
GSNAP + Cufflinks	79.4	71.2	65.7	58.4
GSNAP + Cufflinks (subsample^2^)	80.3	72.7	60.2	66.3
TopHat + Scripture	70.3	67.9	60.8	62.5

^1^Base level accuracy and percentage of confirmed junctions with different combinations of mapper and assembler on the sample ps94 males compared to the orthology annotation and the EST annotation (^2^based on 48 M reads).

To rule out the possibility of sample-specific effects we repeated the entire procedure on a different sample (ps94 females) and obtained very similar results (Table S2).

When comparing the splice junctions recovered by the three approaches we noted a large disparity (Figure S1a). The combination TopHat-Cufflinks resulted in the smallest number of private splice junctions, while TopHat-Scripture had the largest number (and fraction) of splice junctions specific to this approach. An even lower overlap among the approaches was noted on the isoform level (Figure S1b). Between 45.0% (TopHat-Cufflinks) and 84.4% of the isoforms were specific to a single approach.

Next, we asked whether including more samples would increase the accuracy of the transcriptome reconstruction. Intuitively, we expect that it would, as more samples would provide higher coverage for rarely used splice junctions and add new isoforms. To test this idea we merged the mapped reads from all four samples and ran Cufflinks and Scripture. Interestingly, independent of which combinations was used, we did not obtain a higher base- or junction-level accuracy (Table S3). Also changing the settings of Cufflinks to allow for low frequency isoforms (minimum isoform fraction = 0) did not improve the results (data not shown).

**Figure 2:**
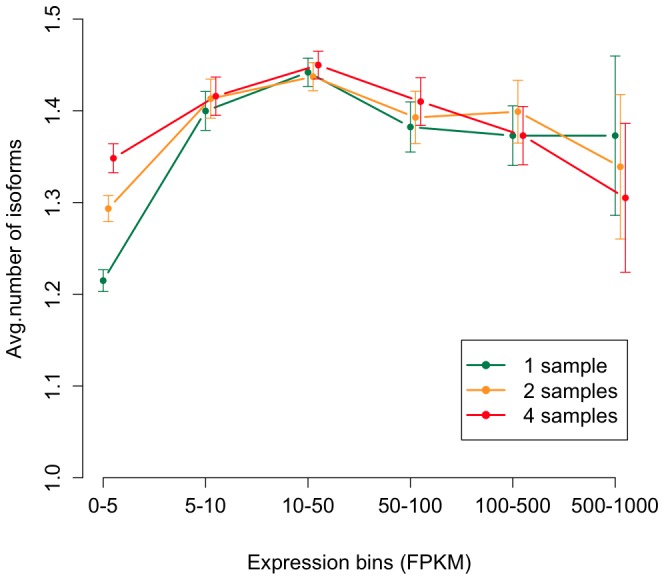
Effect of number of samples and expression intensity on the number of reconstructed isoforms. Isoform reconstruction is performed with TopHat-Cufflinks using one sample (ps94 males), two samples (ps94 males + ps94 females) and all four samples On the x-axis FPKM = Fragments per Kilobase of exon per Million fragments mapped, on the y-axis the average number of reconstructed isoforms is reported. Adding samples increases the amount of predicted isoforms only for low and moderate expression levels.

To investigate the influence of coverage on the reconstruction of isoforms, we measured the total number of reconstructed isoforms using one sample (ps94 males), two samples (ps94 males + ps94 females) or all four samples combined. After grouping the transcripts according to expression intensity, we noted that the combination of multiple samples increased the number of transcripts for lowly expressed genes, while fewer transcripts were discovered in pooled samples of highly expressed genes (Figure 2).

Given that a higher number of mapped reads did not improve the annotation overall, but rather lowered the quality of the annotation (Table S3), we reasoned that increased coverage obtained by merging different samples may reduce the accuracy of isoform deconvolution for Cufflinks, as previously proposed in a comparison of reference-based assemblers [30]. To test this hypothesis, we sampled ∼48 M reads randomly from those mapped by GSNAP in order to equalize this amount to the number of reads mapped by TopHat and then performed the reconstruction with Cufflinks. Indeed, we observed an increase in performance for GSNAP-Cufflinks on the subsample compared to GSNAP-Cufflinks on the whole sample (Table 1). Thus, the performance of both TopHat-Cufflinks and GSNAP-Cufflinks strategies was nearly identical as long as a similar number of mapped reads was supplied to Cufflinks, with a slight advantage of TopHat-Cufflinks.

Our evaluation of reference based RNA-Seq annotation strategies suggests that the choice of mappers is not essential, with TopHat producing slightly better overall annotations than GSNAP. On the other hand, Cufflinks consistently out-performed Scripture. Since the combination TopHat-Cufflinks was the most conservative one, with the highest fraction of splice junctions and isoforms common to all three approaches, we relied on the combination of TopHat and Cufflinks for the remainder of the analyses.

To shed further light on the genes for which we failed to confirm junctions in our data, we performed a GO analysis and detected a significant enrichment (p-value <0.001) for numerous classes related to development, like body morphogenesis or structural constituent of the larval and pupal cuticle, which suggests that unconfirmed genes are mainly expressed in other developmental stages. Other genes for which we failed to confirm junctions fell into the GO category sensory perception of smell and taste. Since these genes are expressed only in few tissues they may not be detectable in our pooled samples (see Materials and methods), as similarly observed in *D. melanogaster* [4].

### Improving the Annotation of the *D. Pseudoobscura* Reference Genome

Rather than relying on a single RNA sample, we used four different RNA samples for our annotation; male and female adults from two inbred strains (ps94 and ps88). We ran TopHat-Cufflinks on each sample independently, retaining only transcripts that were detected in at least two of the four samples (see Materials and methods) in order to minimize spurious transcripts and assembly artifacts generated by Cufflinks. This conservative filtering step discarded about 50% of the transcripts in each of the samples. Expression level of the discarded transcripts is significantly lower than of transcripts confirmed in two samples (Mann-Whitney test: p-value <2.2×10^−16^). While this observation suggests that this method results in exclusion of some true isoforms with low expression levels, we nevertheless chose to exclude sample specific isoforms to reduce assembly artifacts.

Our analysis showed that 21.5% of the retained transcripts are male-specific and 10.4% are female-specific. The number of strain-specific transcripts (about 6%) does not exceed chance effects, approximated by the proportion of transcripts specific to two samples with discordant sex and strain (data not shown). These findings are consistent with a higher correlation in gene expression between different strains than between sexes [32]. We note that the proportion of male *vs.* female-specific transcripts is not directly comparable to the fraction of sex-biased genes typically found in the literature [32], [33] since we report sex specific transcripts rather than comparing the expression intensity.

We compared our new *D. pseudoobscura* annotation to the FlyBase annotation (see Materials and Methods) (Table 2) on six different levels: gene, transcript, exons, CDS and UTR levels, and discuss each in turn below.

**Table 2:** Comparison of our annotation *vs.* the FlyBase annotation r2.23.

Genes	FlyBase r2.23	Our annotation
Total number of genes	16074	16743
Total number of genes + strand	8009	8325
Total number of genes – strand	8065	8357
Total number of genes no strand	0	61
Mean gene length (bp)	3538.30	4554.39
Gene density gene/Mb	105.24	109.62
Number of transcripts	16619	23605
Average isoforms per gene^1^	1.03	1.41
Percent of transcripts with introns	75.89	81.41
Mean transcript length	1484.66	2175.79
**Exons** 2	**FlyBase r2.23**	**Our annotation**
Number	57535	59213
Mean number per transcript	3.58	3.54
GC content	53.54	51.82
Mean length (bp)	406.63	566.04
Total length (bp)	23395488	33517186
**Introns** 2	**FlyBase r2.23**	**Our annotation**
Number	41461	42470
Mean number per transcript	3.44	3.44
GC content	41.85	41.57
Mean length (bp)	797.30	897.53
Total length (bp)	33056749	38118309
**UTRs^3^**	**FlyBase r2.23**	**Our annotation**
Number of genes having UTR	577	7066
Mean UTR length (bp)	215.09	769.57
Number of 5′UTRs	445	6695
Mean 5′UTR length (bp)	149.76	498.49
Number of 3′UTRs	493	6619
Mean 3′UTR length (bp)	274.06	1043.77

^1^only considering multiple exon genes,

^2^only for the longest isoforms of each gene.

### Annotation of Genes

**Figure 3:**
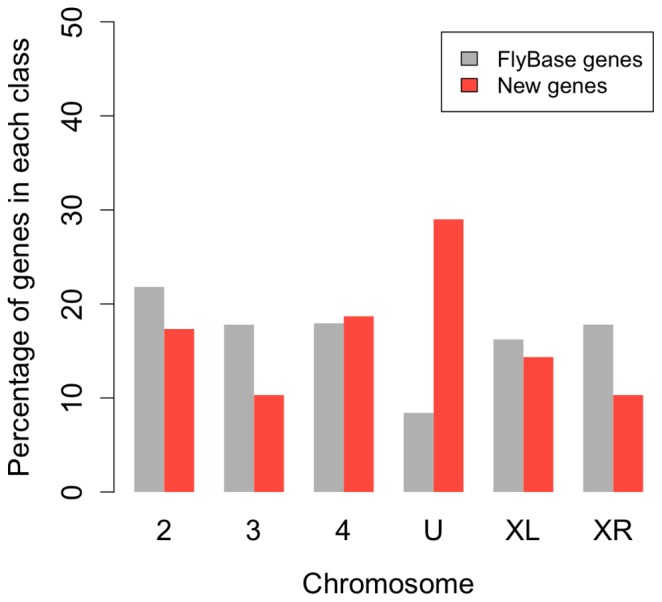
Chromosomal distribution of new genes. Most of the new genes are located on the unassembled contigs (U).

We extended 6523 (40%) FlyBase gene models, of which 545 were extended only at the 5′, 421 only at the 3′ and 5557 at both, 5′ and 3′-termini. On average a gene model was extended by 2.8 kb. We discovered a total of 669 genes not included in the FlyBase annotation (new genes) (Table S4): 116 of these are located in introns of FlyBase genes and 553 are intergenic. We observed splicing evidence for 303 (45%) new genes. About one third of the new genes are located on unassembled contigs (referred as U) (Figure 3). The average length of newly annotated genes is 1.3 kb. Overall, new genes are significantly shorter (Mann-Whitney test: p-value <3.525×10^−12^), less GC rich (Mann-Whitney test: p-value <2.2×10^−16^), have fewer isoforms (Mann-Whitney test: p-value <2.2×10^−16^) and a lower expression level (Mann-Whitney test on ps94 males: p-value <2.2×10^−16^– Figure S2) when compared to FlyBase genes. The splice-site composition for new genes was very similar to FlyBase genes (Figure S3). Since TopHat recognizes only canonical splice junctions, it is not clear to what extent this result reflects a true biological signal or the properties of TopHat. We asked whether the new genes have orthologs in *D. melanogaster* using BLASTX with a stringent e-value of 10^−10^. While we found 56 genes with a hit to *D. melanogaster* proteins, 31 of them did not yet have a FlyBase annotated ortholog (one-to-one orthologs). These genes were probably missed in the initial orthology annotation of *D. pseudoobscura* [16]. Moreover, almost 50% (14 genes) of the one-to-one orthologs are located on the “U” contigs, underlining their incomplete annotation. A Gene-Ontology (GO) analysis of the one-to-one orthologs showed no enrichment for any particular category. The small fraction of one-to-one orthologs suggests that most of the new genes might be specific to the *D. pseudoobscura* lineage, although more systematic comparisons are necessary to exclude the existence of orthologs in other species. Finally, we classified new genes as coding/non-coding using the Coding Potential Calculator (CPC), which has been shown to be highly accurate for *D. melanogaster* genes [34]. Only 53 genes (8%) showed coding evidence on at least one strand. While this may suggest that a large fraction of the new genes may be non-coding, we caution that out of the 56 new genes for which we identified an ortholog in *D. melanogaster*, only seven were classified as coding by the CPC software.

### Annotation of Transcripts

**Figure 4:**
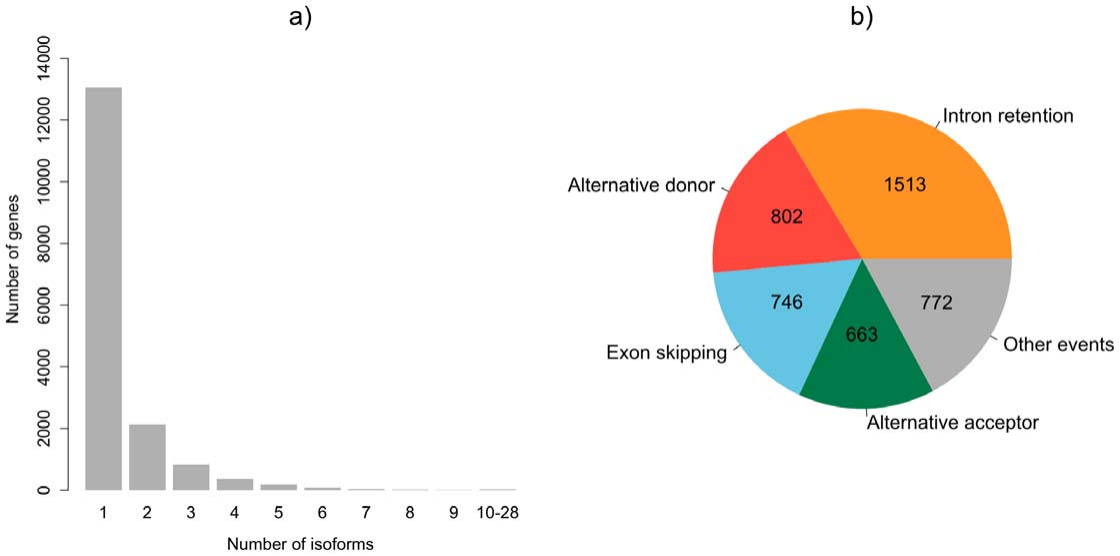
Alternative splicing in *D. pseudoobscura*. a) Distribution of number of isoforms per gene, b) Distribution of alternative splicing modes.

At the transcript level we identified 6986 novel (previously unannotated) isoforms, of which 6232 are associated to 3480 FlyBase genes and 754 are novel isoforms for the 669 newly annotated genes. The distribution of novel transcripts across samples is reported in Table S6. We increased the number of genes with alternative splicing from 339 (3%) in the *D. pseudoobscura* annotation release 2.23 to 3233 (30%) in our updated annotation, which corresponds to 4496 new alternative splicing events. On average, we detected 1.6 isoforms per gene. While most genes have only a single isoform, some genes have a large number of annotated isoforms (Figure 4a). We classified the 4496 newly discovered alternative splicing events into different alternative splicing modes using the online tool ASTALAVISTA (Figure 4b). More than one third of the alternatively spliced events are due to intron retentions, and the rest is almost equally distributed among the other modes (i.e. alternative acceptor, alternative donor, exon-skipping).

**Figure 5:**
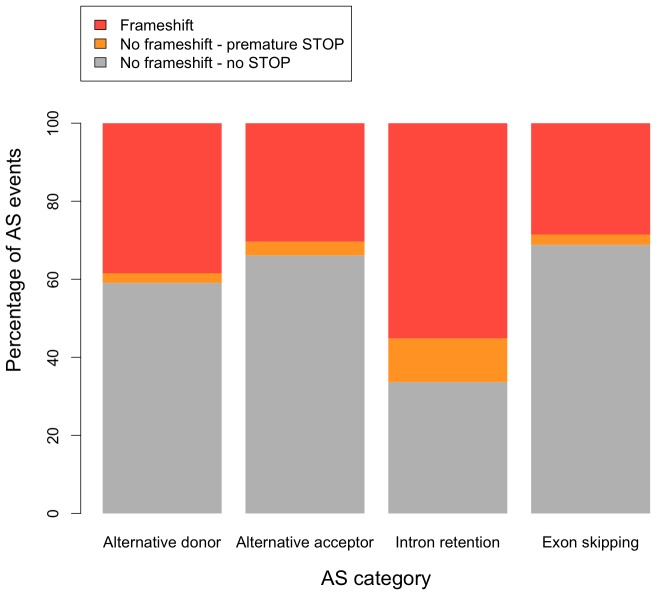
Effects of alternative splicing events on the coding sequence (CDS) in *D. pseudoobscura*. Stop codons are rare in all alternative splicing (AS) modes. Frameshifts show a more heterogeneous distribution, with intron retention events harboring most frameshifts.

To validate the inferred isoforms we tested for preservation of the open reading frames and the introduction of premature stop codons in the alternatively spliced region. Premature stop codons are rare which is consistent with the expectation that the probability of obtaining a stop codon in the alternatively spliced regions is low since these regions are on average very short (avg. length alt. donors = 123±219 bp, avg. length alt. acceptors = 101±359 bp, avg. length intron retentions = 147±299 bp, avg. length exon-skipping = 251±475 bp). Reading frame conservation is highly heterogeneous among alternative splicing modes, with exon-skipping events having the fewest and intron retentions having the highest number frameshifts and premature stops (Figure 5). This result could be caused either by a higher fraction of unprocessed RNAs or by an artifact introduced by our annotation procedure, as Cufflinks might erroneously merge adjacent exons if they are very close to each other. However, when we compare the patterns of frameshift and premature stops for different alternative splicing modes in the *D. melanogaster* annotation (release 5.43) and against a recent *D. melanogaster* annotation obtained from RNA-Seq (named mb8) [35], the distribution of frameshifts and premature stops is very similar to *D. pseudoobscura* (Figure S4). This suggests that the observed distribution is not an artifact of our annotation procedure.

We performed a GO analysis to test for an enrichment of particular functional categories among the different alternative splicing modes. For intron retention and alternative acceptor no GO category was enriched. Alternative donors are enriched for genes involved in transmembrane transport (p-value = 0.006), substrate-specific transporter activity (p-value = 0.014), transporter activity (p-value = 0.026), transmembrane transporter activity (p-value = 0.027) and substrate-specific transmembrane transporter (p-value = 0.027). Finally, for exon-skipping events numerous categories are overrepresented, the most significant of them (p-value <0.001) relating to actin filament-based process, actin cytoskeleton organization, plasma membrane, regulation of multicellular organismal process, developmental process, biological regulation and regulation of biological process (all with p-value <0.001). The complete list of GO categories is reported in Dataset S1. Enrichment maps uncovering the relationships among the overrepresented GO terms are given in Figure S5.

We observed the presence of RNA polymerase run-on fragments [36] for 168 genes, as reported by cuffcompare (class-code “p”). These are presumably transcripts in which the RNA Pol II continued transcription for some distance beyond the normal 3′end of the gene as previously shown in yeast [36]. We asked if particular classes of genes are more prone to produce run-on fragments by performing a GO analysis and found a significant enrichment for biological regulation (p-value = 0.01) and signal transduction (p-value = 0.043).

### Annotation of Exons

At the exon level we extended 15539 terminal exons previously annotated in FlyBase, and added 19682 novel exons. Among the novel exons, 3809 were added at the 5′end, 546 at the 3′end and 15327 were added internally. It is not surprising that we discover more exons at the 5′- compared to the 3′-end since exons in 5′ are more difficult to predict by in silico annotation [37], because they are often flanked by long introns. To test for purifying selection operating on novel exons we compared the PhastCons conservation scores of novel exons and introns (see Materials and methods and Figure S6). We found that the novel exons were more conserved than introns (Mann-Whitney test: p-value <2.2×10^−16^) or short introns, which are a good proxy for neutrally evolving sequences [29] (Mann -Whitney test: p-value <2.2×10^−16^). This higher conservation relative to presumably neutral sequences suggests that novel exons are subject to selective constraints and thus putatively functional.

### Annotation of Coding Sequences and UTRs

At the CDS level we predicted 6104 complete ORFs, of which 5568 are associated with novel isoforms of 3201 FlyBase genes and 536 with isoforms of 476 new genes.

At the UTR level we annotated UTRs for 7066 (42%) genes. On average, 5′UTRs increase in length by 500 bp whereas 3′UTRs increase by more than 750 bp (Table 2). Our findings represent a major improvement compared to the FlyBase annotation 2.23, in which UTRs are present for only 2% of the genes. Compared to *D. melanogaster* (FlyBase release 5.43), UTRs are significantly longer in *D. pseudoobscura*, regardless of whether the FlyBase or mb8 annotation [35] is used (FlyBase: Mann-Whitney test on 5′UTRs: p-value <2.2×10^−16^, Mann-Whitney test on 3′UTRs: p-value <2.2×10^−16^; mb8 annotation [35]: Mann-Whitney test on 5′UTRs: p-value <2.2×10^−16^, Mann-Whitney test on 3′UTRs: p-value <2.2×10^−16^) (Figure S7). This probably reflects the larger size of the *D. pseudoobscura* genome. Length differences between 5′ and 3′UTRs are comparable with *D. melanogaster*, where 3′UTRs are significantly longer than 5′UTRs (Mann-Whitney test: p-value <2.2×10^−16^) due to higher abundance of regulatory elements in 3′UTRs [38].

Previous analyses based on coding sequences alone found no significant differences in the length of first and terminal introns in *D. pseudoobscura* [39]. Since this contrasted with other work suggesting longer first introns in *D. melanogaster* [40], we hypothesized that introns located in the 5′UTR may be responsible for this discrepancy. Using only genes for which we have improved the annotation, we found that first introns (intron at first position independent of whether they are located in UTRs or not) are longer than internal (Mann-Whitney test: p-value <2.2×10^−16^) and terminal introns (Mann-Whitney test: p-value <2.2×10^−16^). These results are now consistent with the pattern observed in *D. melanogaster* [40], supporting the hypothesis that longer first introns are a general patterns of eukaryotic gene structures. Introns located in the 5′UTR are significantly longer than introns in coding sequence and in the 3′UTR (Mann-Whitney test: p-value <2.2×10^−16^) (Figure S8), which is again in accordance with the pattern in *D. melanogaster* [41]. The proposed explanation for this pattern invokes negative selection against intron contraction events in 5′UTRs due to the potential introduction of deleterious upstream start codons in 5′UTR exons [41].

When comparing exon length we observed that first and last exons are both significantly longer than internal exons (first *vs.* internal: Mann-Whitney test: p-value <2.2×10^−16^; last *vs.* internal: Mann-Whitney test: p-value <2.2×10^−16^), most likely due to reduced selective constraints on UTRs. This pattern is also consistent with *D. melanogaster* (Mann-Whitney test: first exons *vs.* internal exons: p-value = 2.6×10^−8^, internal exons *vs.* terminal exons: p-value <2.2×10^−16^, first exons *vs.* terminal exons: p-value <2.2×10^−16^).

In summary, these results suggest that factors governing overall gene structure are similar between *D. melanogaster* and *D. pseudoobscura*.

### *De Novo* Assembly

We performed *de novo* transcriptome assembly to discover genes not included in the published genome scaffolds. We identified 99 candidates, referred as new-extra genes. Like for other newly discovered genes, we required new-extra genes to be expressed in at least two samples. Compared to new genes located on the genome scaffolds, new-extra genes are significantly shorter at the transcript level (Mann-Whitney test: p-value <1.1×10^−8^) and have lower GC content (Mann-Whitney test: p-value <1.7×10^−8^). We validated these genes using different approaches. First, like for novel exons, we evaluated if purifying selection was acting on them, and found that exons of new-extra genes are significantly more conserved than introns (p-value <2.2×10^−16^). Second, we tested for sequence conservation between *D. pseudoobscura* and *D. melanogaster* or *D. persimilis* using BLASTN, BLASTX and Exonerate (see Materials and methods). 34 orthologs were detected in *D. melanogaster* and 85 in *D. persimilis* by at least one of the three methods. We detected orthologs for 92 of the 99 new-extra genes by at least one of the three methods (34 in *D. melanogaster* and 85 in *D. persimilis*). Of the 92 orthologs, 19 were classified as coding by CPC. The remaining seven genes may be either *D. pseudoobscura*-specific or fast evolving. One of these genes was also classified as coding by CPC, resulting in a similar fraction as for genes with an ortholog, suggesting no difference in coding potential. Detailed information about length, GC content, coding potential and orthology assignments for new-extra genes is reported in Table S5. Sequences of new-extra genes in FASTA format are given in Dataset S2.

In addition, we used the orthology annotation of the FlyBase genes (see Materials and Methods) to evaluate the accuracy of Trinity regarding transcriptome reconstruction and observed a significantly lower accuracy for Trinity (Text S1) compared to the reference-based assemblers.

## Discussion

A comparison of different mappers and assemblers allowed us to evaluate different reference-based strategies for genome annotation from RNA-Seq reads. We found, surprisingly, that the use of a more accurate mapper like GSNAP does not improve the accuracy of transcriptome reconstruction. Since GSNAP maps more reads compared to TopHat, increased coverage for highly expressed genes can be an obstacle to the correct reconstruction of isoforms, as shown in [30]. As the per-run coverage of sequencing technologies is rapidly increasing, solutions will be needed to cope with this issue. One likely advantage of including more reads would be the improved annotation of lowly expressed genes, but we did not test this in our analysis. Instead, our quality assessment only relied on an overall performance.

Additionally, merging reads from different samples did not improve the reconstruction independently of the method used. This observation is consistent with recent guidelines, which suggest performing transcriptome reconstruction independently for each sample [42]. This phenomenon can be attributed to the complexity of disentangling isoforms when the assembler is faced with the computational burden of handling many reads, a pitfall for both assemblers tested here, Cufflinks and Scripture.

We found Cufflinks to be more accurate than Scripture. This observation is in contrast to the results found for simulated data from mouse [30], where the opposite pattern was detected. Further investigation is necessary to understand the nature of this discrepancy.

A striking observation was the low consistency among the three approaches evaluated here. Irrespective of whether we analyzed the number of shared splice junctions or isoforms, we noted that each mapper-assembler combination resulted in a high fraction of private annotations, i.e. annotations specific to one approach (from 45% of the isoforms for TopHat-Cufflinks to 84% for TopHat-Scripture). These private annotations likely represent an annotation artifact. A similar amount of private isoforms was also found when Cufflinks and Scripture were applied to RNA-Seq data from human and mouse [30].

A high number of false positives produced by reference-based assemblies has been noticed before for Cufflinks [30], [43] and Scripture [30]. We tried to reduce these effects by only using transcripts, which occurred in at least two samples. Even using this conservative criterion we identified alternative splicing for more than 30% of the genes. On the other hand, this is still a considerably lower number than has been detected in *D. melanogaster* (30% in [4], 60% in [35]). The difference can be explained since we only used adult flies in our study; most likely, the number of alternatively spliced genes will dramatically increase when additional developmental stages are included and isoforms not expressed in adults can be analyzed.

Among different alternative splicing modes, intron retention seems to be predominant in *D. pseudoobscura*. The high fraction of frameshifts and premature stop codons and the short length of retained introns suggest that most of them may be unprocessed mRNAs. On the other hand, the pattern of exon length, intron length and UTR length are similar to those found in *D. melanogaster* [41], suggesting phylogenetic inertia or similar selective pressures maintaining the gene length in both species.

Our *de novo* assembly recovered 99 candidate transcripts potentially belonging to genes not yet included in the current *D. pseudoobscura* annotation. New-extra genes have reduced GC content compared to new genes located on the scaffolds, most likely related to their very low expression level [44]. This may suggest that new-extra genes are presumably located in heterochromatin, which is notoriously difficult to assemble. Moreover, the finding of new-extra genes highlights the incompleteness of the current *D. pseudoobscura* assembly and raises further questions about the true number of genes in poorly annotated genomes.

### Conclusions

We show that the annotation of genomes can be improved by using RNA-Seq data in presence of a reference genome. Nevertheless, our analyses also demonstrated that RNA-Seq based annotation is still in its infancy and more reliable approaches need to be developed. Using RNA-Seq data obtained from adult flies we considerably improved the annotation of *D. pseudoobscura* by revealing almost 7000 new isoforms for 30% of the multiple-exon genes, extending more than 40% of the gene boundaries and discovering 768 novel genes. Multiple conditions and different developmental stages will be needed to dissect the alternative splicing landscape at a deeper resolution. This improved annotation will contribute to the understanding of the regulation of alternative splicing in *Drosophila*. Further studies of putative *D. pseudooobscura*-specific genes may also shed light on genes contributing to speciation and genes with a novel adaptive role in this species.

## Supporting Information

Figure S1 **Common junctions between reference-based approaches.** Venn diagrams of common splice junctions (a) and common isoforms (b) among three annotation methods applied to the sample ps94 males.
(TIFF)

Figure S2 **Expression of FlyBase genes and new genes.** Expression of FlyBase genes *vs.* newly discovered genes in the sample ps94 males and ps94 females.
(PDF)

Figure S3 **Splice site composition of FlyBase genes and new genes.** a) 5′splice-site composition of FlyBase genes, b) 3′splice-site composition of FlyBase genes, c) 5′splice-site composition of new genes, d) 3′splice-site composition of new genes.
(PDF)

Figure S4 **Effect of alternative splicing on the CDS in** ***D. melanogaster.*** a) Using the annotation r5.47, b) Using the *mb8* annotation from [35].
(PDF)

Figure S5 **Enrichment maps for overrepresented GO terms of different alternative splicing modes.** a) Alternative donor AS events, b) Exon-skipping AS events.
(PDF)

Figure S6 **Distribution of PhastCons scores for different annotation features.**
(PDF)

Figure S7 **Length of UTRs in** ***D. pseudoobscura*** **and** ***D. melanogaster.*** **Length comparison of 5′UTRs** ***vs.*** **3′UTRs.**
(PDF)

Figure S8 **Intron length in different parts of the gene in** ***D. pseudoobscura*** **and** ***D. melanogaster.*** Length comparison of introns located in 5′UTRs *vs.* CDS *vs.* 3′UTRs for *D. pseudoobscura* (a) and *D. melanogaster* (b).
(PDF)

Table S1 **Mapping statistics.** Proper pairs are defined as paired-end reads for which both mates are mapped to the reference genome.
(DOC)

Table S2 **Comparison of reference-based approaches for the sample ps94 females.** Base level accuracy and percentage of confirmed junctions with different combinations of mapper and assembler on the sample ps94 females compared to the orthology annotation and the EST annotation (see Results).
(DOC)

Table S3 **Comparison of reference-based approaches for all the samples merged.** Base level accuracy and percentage of confirmed junctions with different combinations of mapper and assembler for all the samples merged compared to the orthology annotation and the EST annotation (see Results)
(DOC)

Table S4 **Table with new genes and their annotation properties.**
(XLS)

Table S5 **Table with new-extra genes and their annotation properties.** The first five characters of the gene_id contain the strain from which the longest version of the gene was extracted (for details see Materials and Methods).
(XLS)

Table S6 **Distribution of novel transcripts across samples.**
(XLS)

Dataset S1 **GO categories for different alternative splicing modes.**
(TXT)

Dataset S2 **FASTA sequences of new-extra genes.**
(TXT)

Dataset S3 **Improved annotation of** ***D. pseudoobscura*** **in GTF format.**
(ZIP)

Text S1 **Evaluation of Trinity on the FlyBase genes.**
(DOC)
