# Supplementary figures and images for: Evaluation of Different Reference Based Annotation Strategies Using RNA-Seq – A Case Study in Drososphila pseudoobscura
Source: PLoS One. 2012 Oct 3;7(10):e46415. doi: 10.1371/journal.pone.0046415 (PMC3463616; doi:10.1371/journal.pone.0046415)

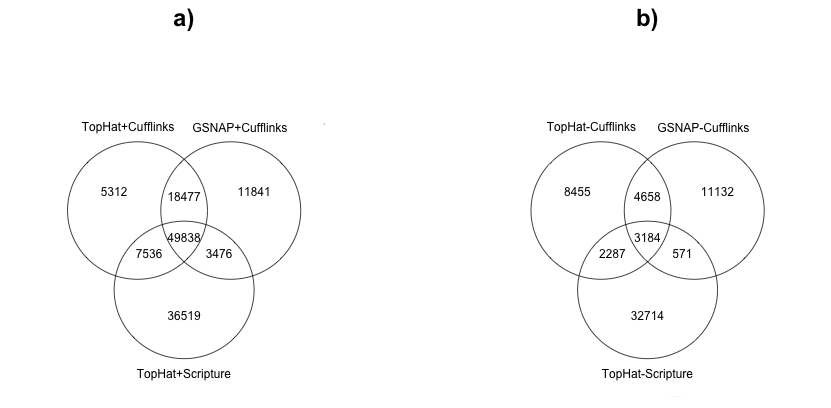

Supplement: Figure S1 — Common junctions between reference-based approaches. Venn diagrams of common splice junctions (a) and common isoforms (b) among three annotation methods applied to the sample ps94 males. (TIFF) [file pone.0046415.s001.tiff]

**a)**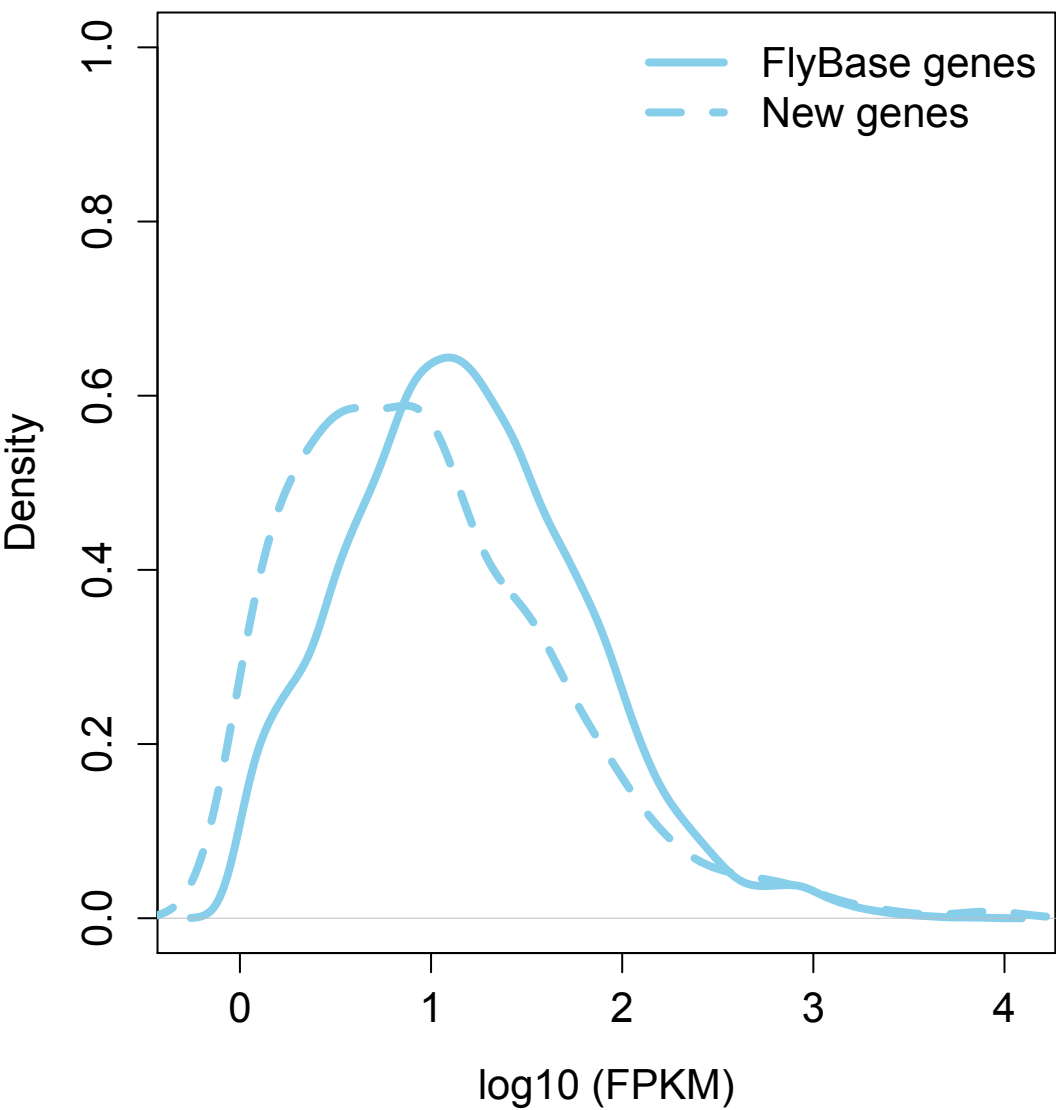**b)**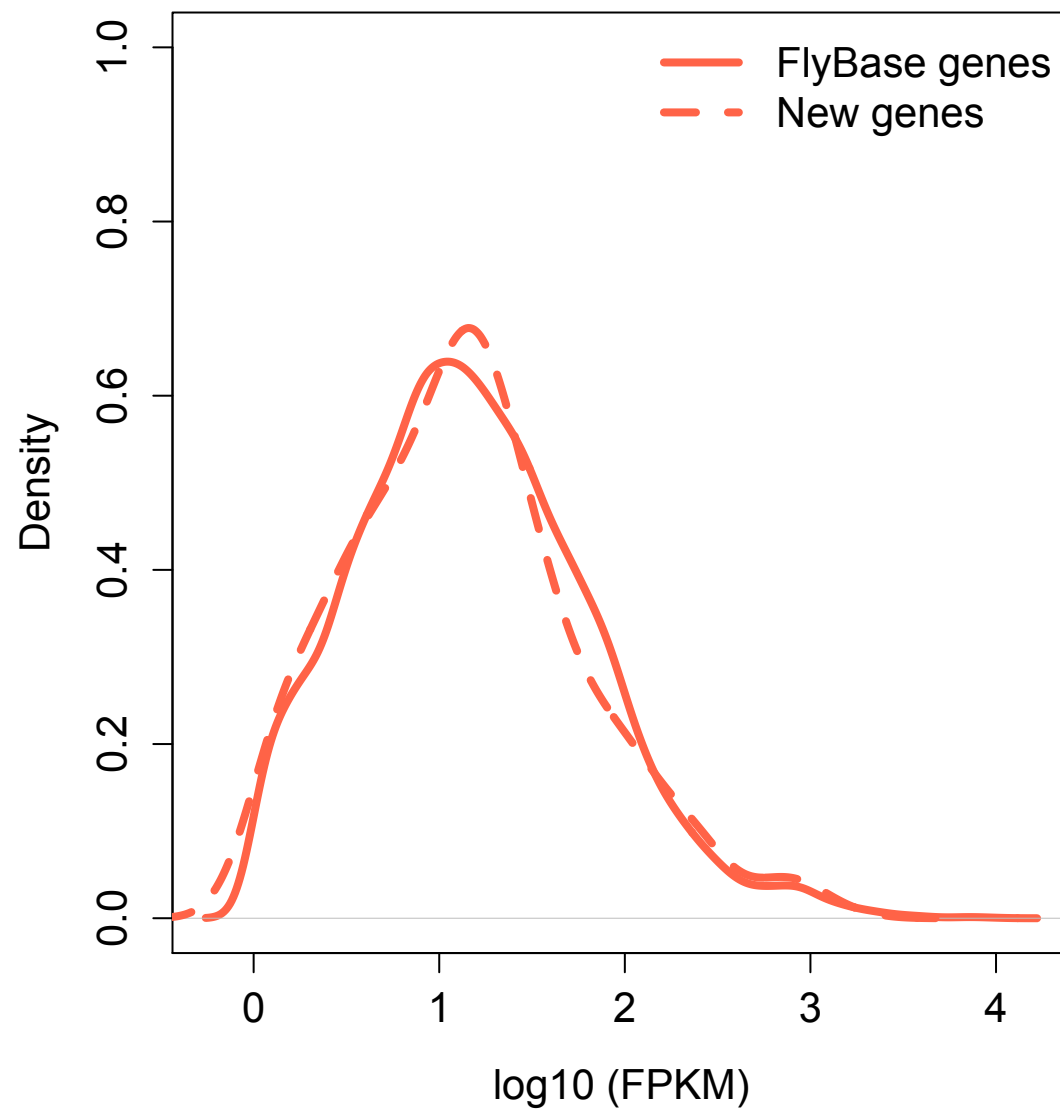

Supplement: Figure S2 — Expression of FlyBase genes and new genes. Expression of FlyBase genes vs. newly discovered genes in the sample ps94 males and ps94 females. (PDF) [file pone.0046415.s002.pdf]

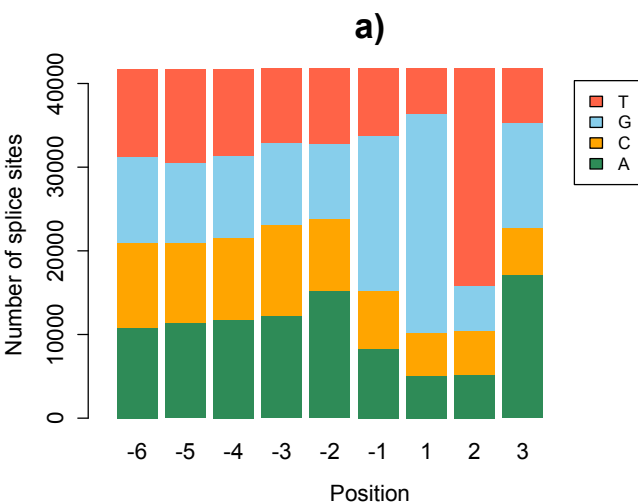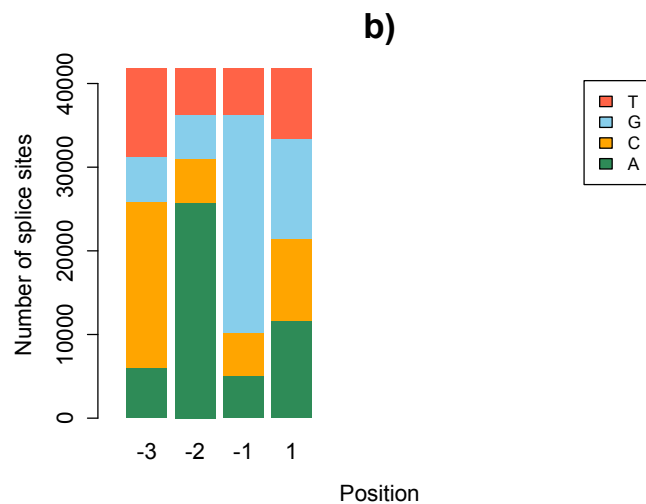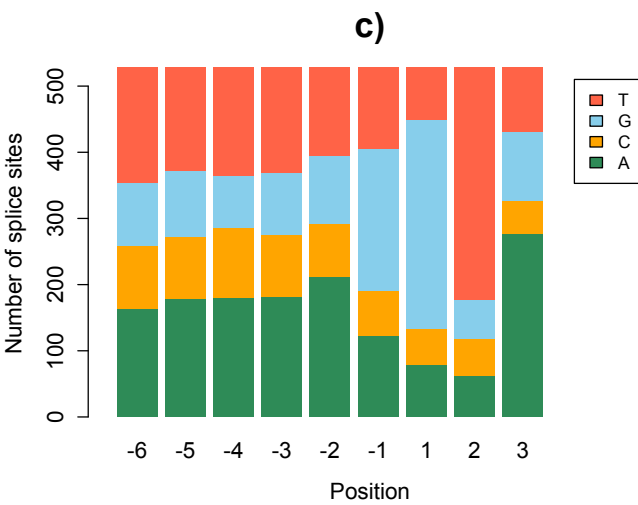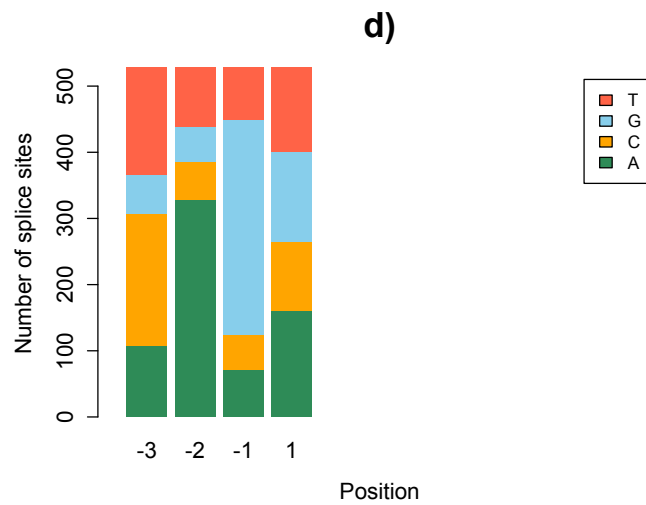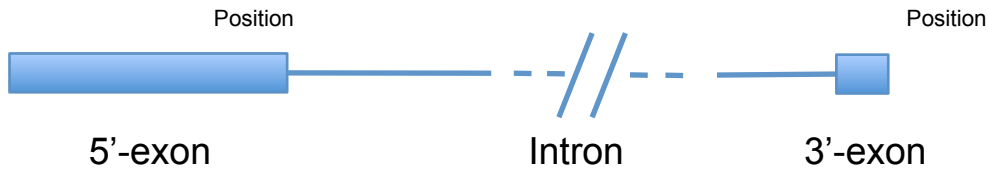

Supplement: Figure S3 — Splice site composition of FlyBase genes and new genes. a) 5′splice-site composition of FlyBase genes, b) 3′splice-site composition of FlyBase genes, c) 5′splice-site composition of new genes, d) 3′splice-site composition of new genes. (PDF) [file pone.0046415.s003.pdf]

**a)**

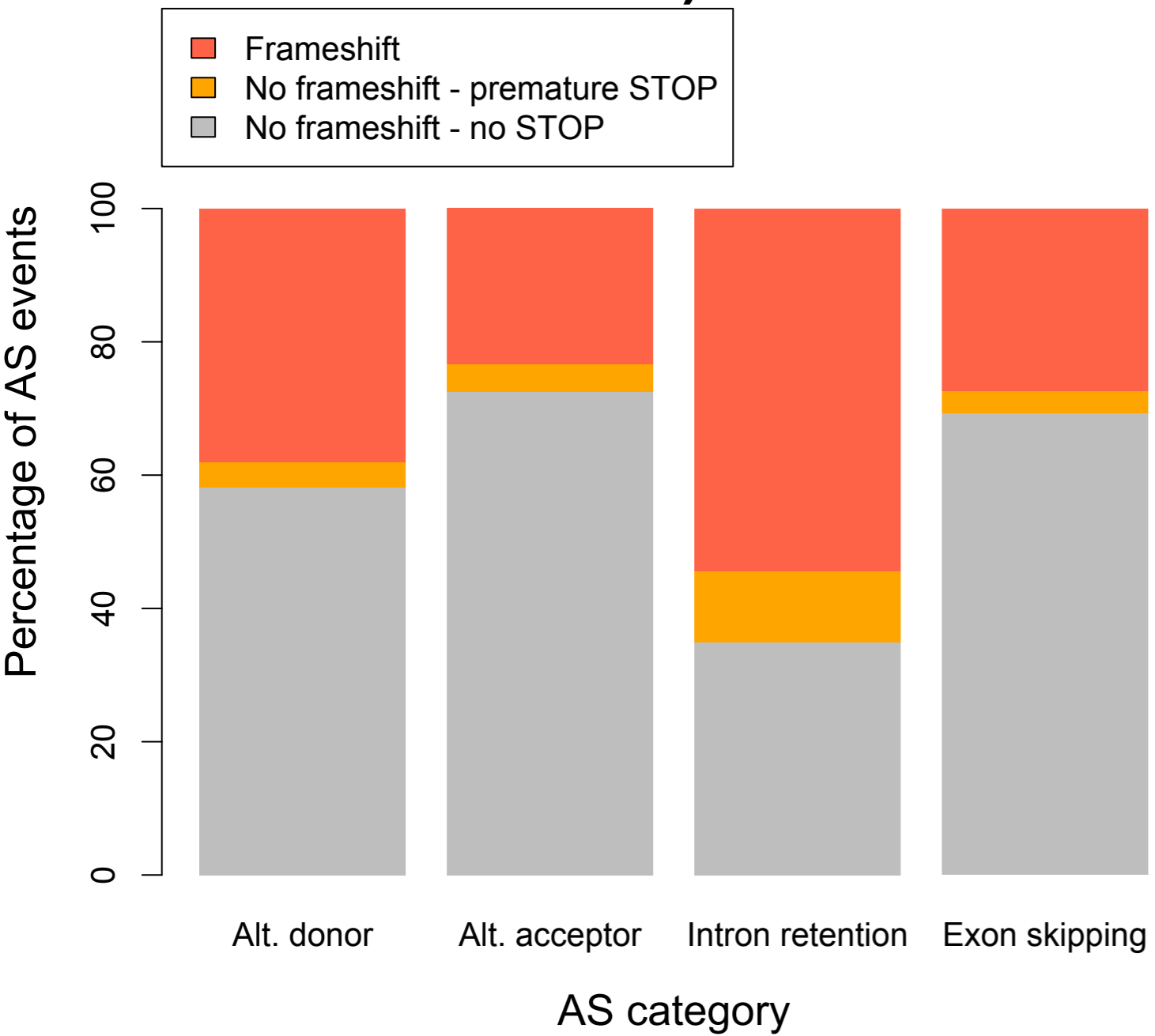

**b)**

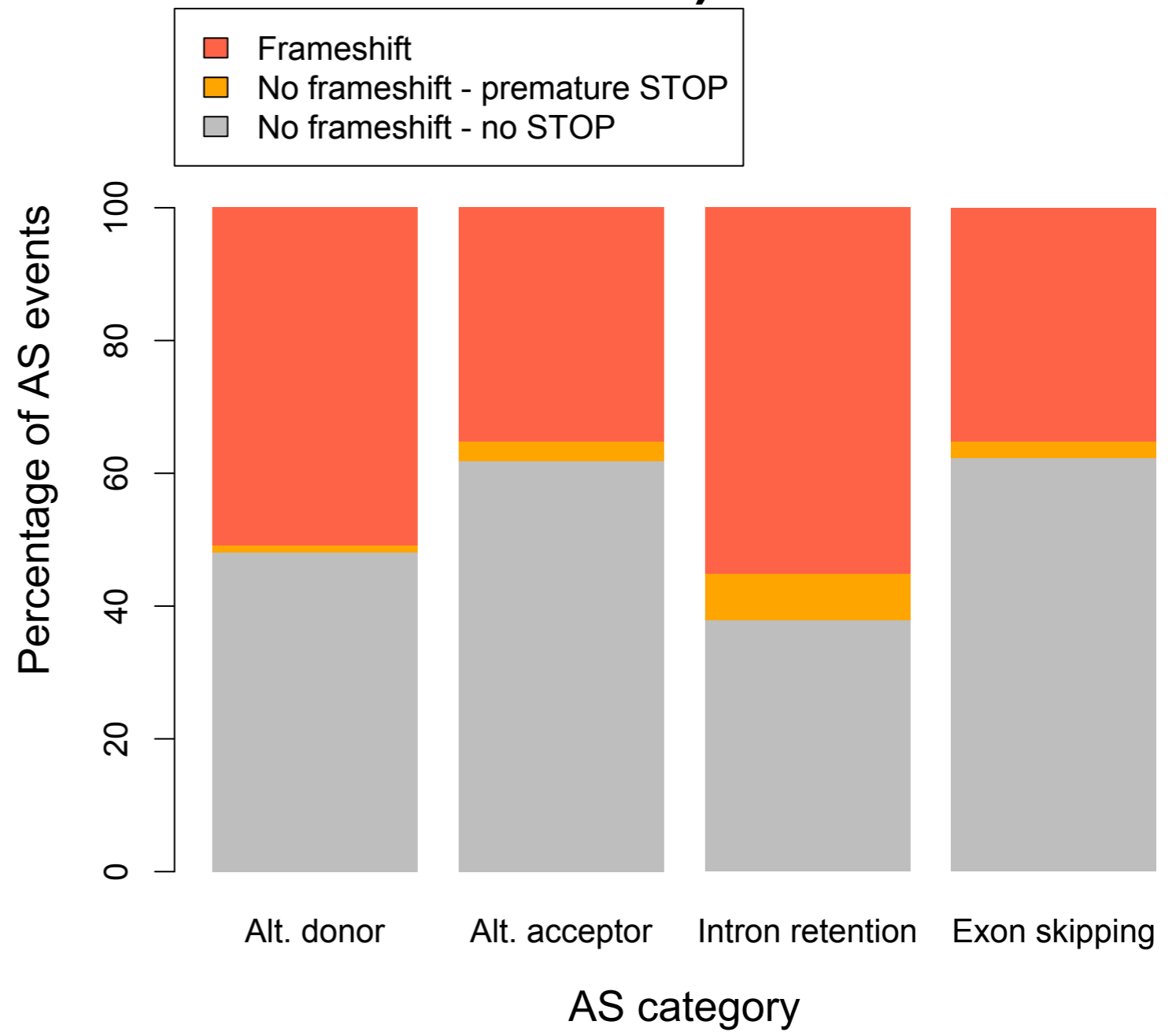

Supplement: Figure S4 — Effect of alternative splicing on the CDS in D. melanogaster. a) Using the annotation r5.47, b) Using the mb8 annotation from [35]. (PDF) [file pone.0046415.s004.pdf]

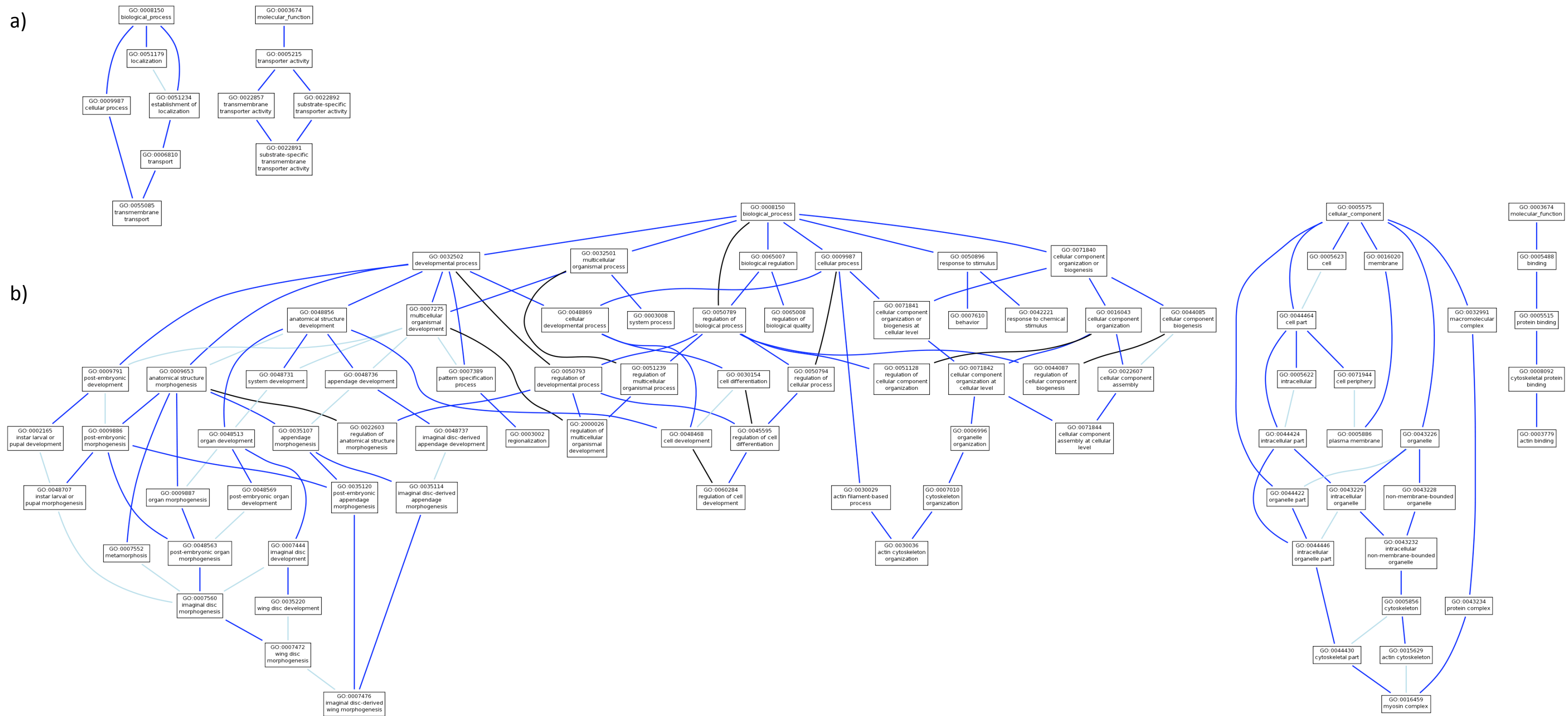

Supplement: Figure S5 — Enrichment maps for overrepresented GO terms of different alternative splicing modes. a) Alternative donor AS events, b) Exon-skipping AS events. (PDF) [file pone.0046415.s005.pdf]

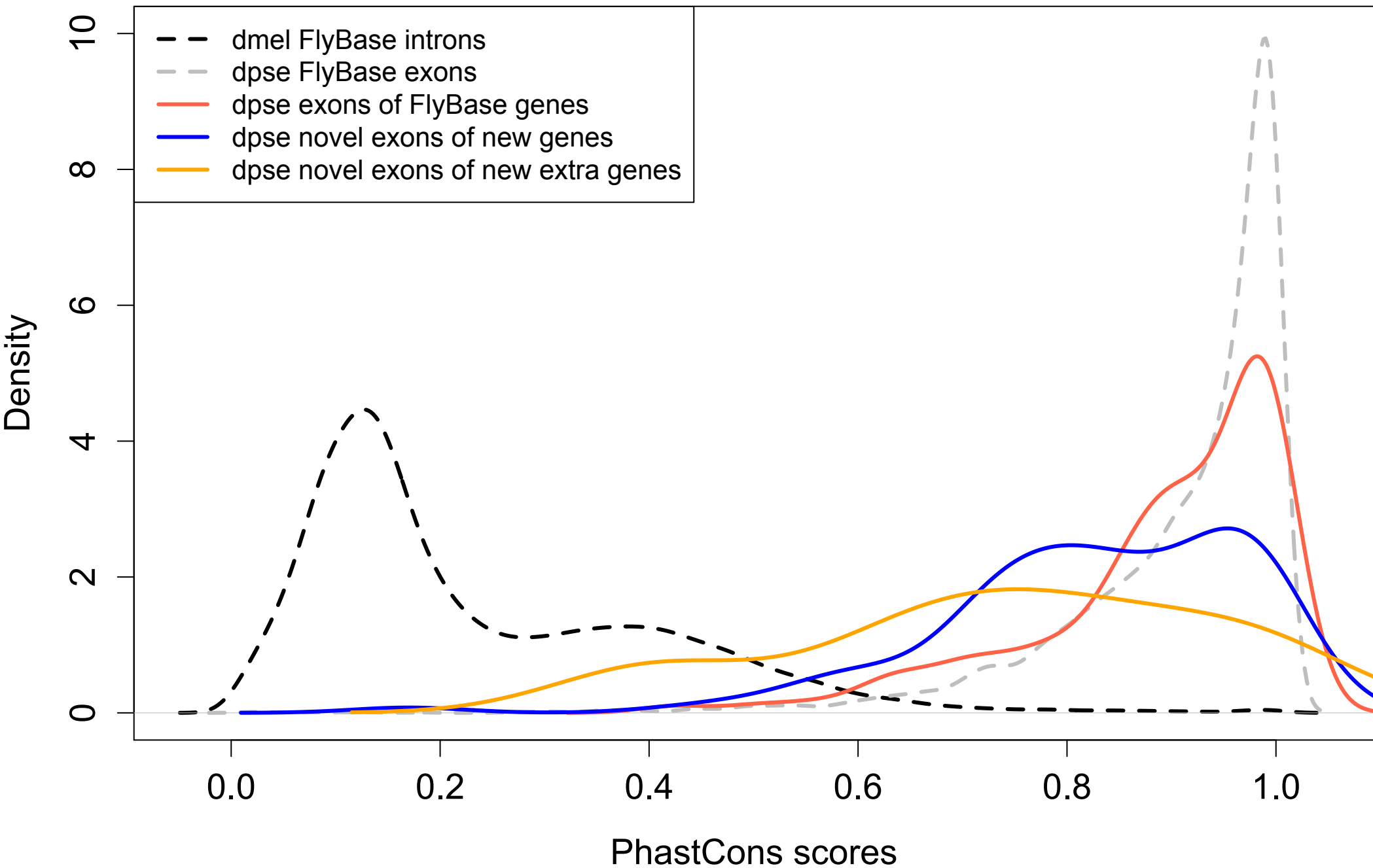

Supplement: Figure S6 — Distribution of PhastCons scores for different annotation features. (PDF) [file pone.0046415.s006.pdf]

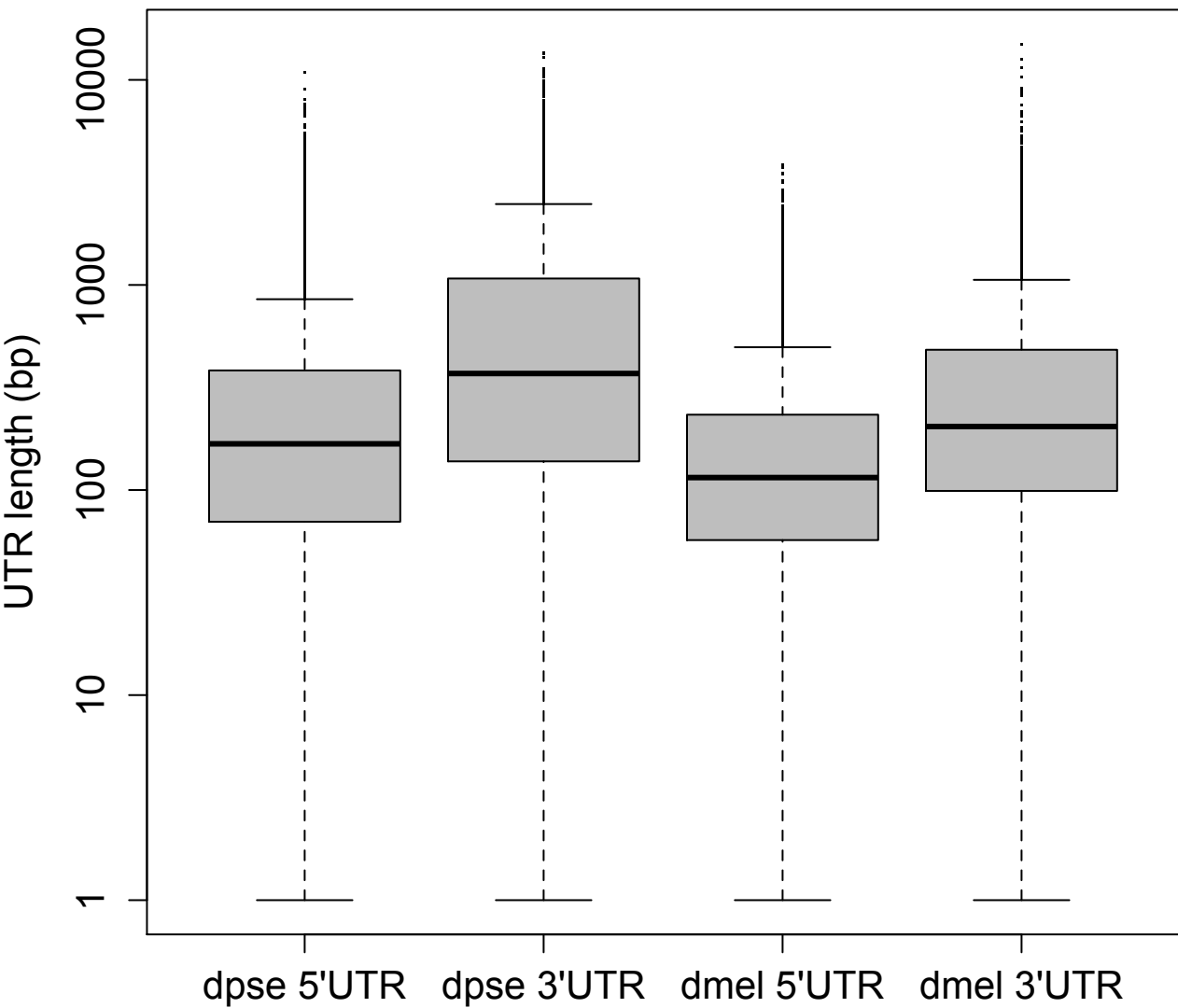

Supplement: Figure S7 — Length of UTRs in D. pseudoobscura and D. melanogaster. Length comparison of 5′UTRs vs. 3′UTRs. (PDF) [file pone.0046415.s007.pdf]

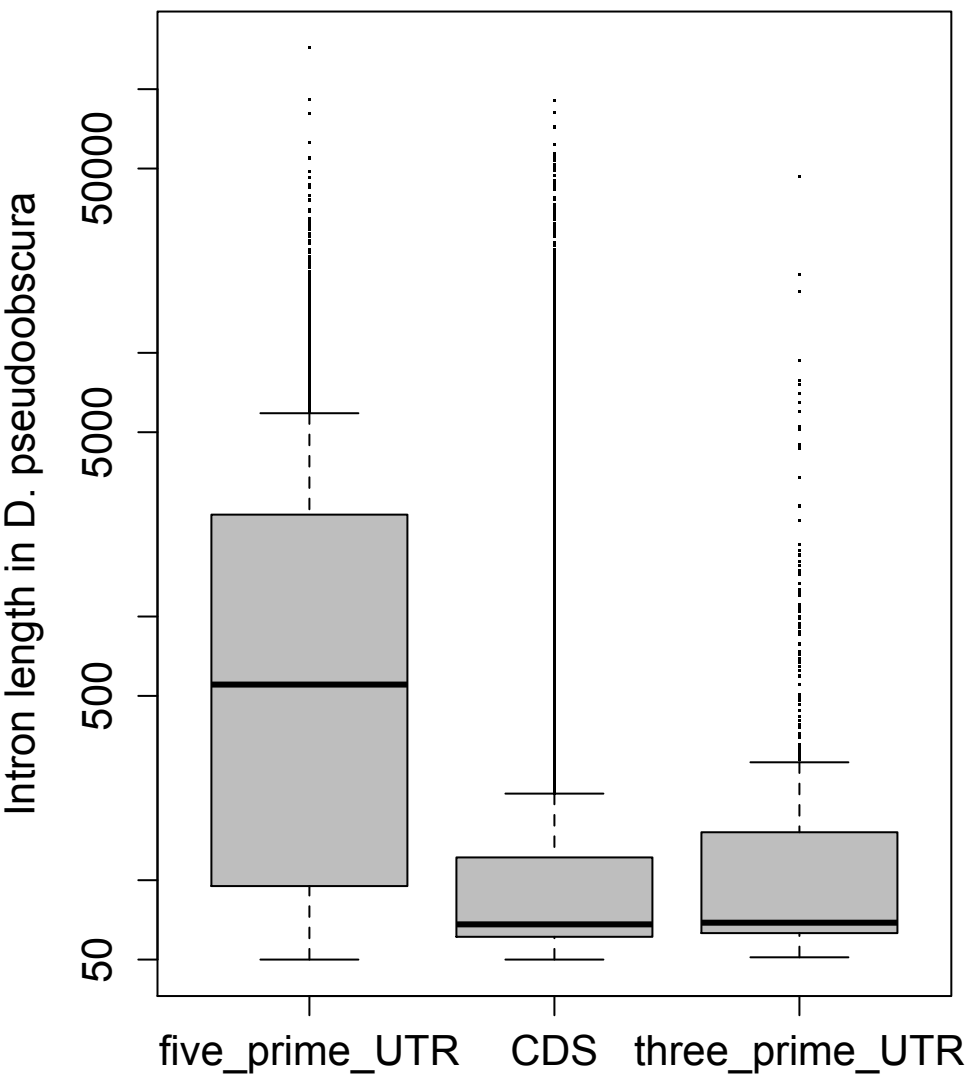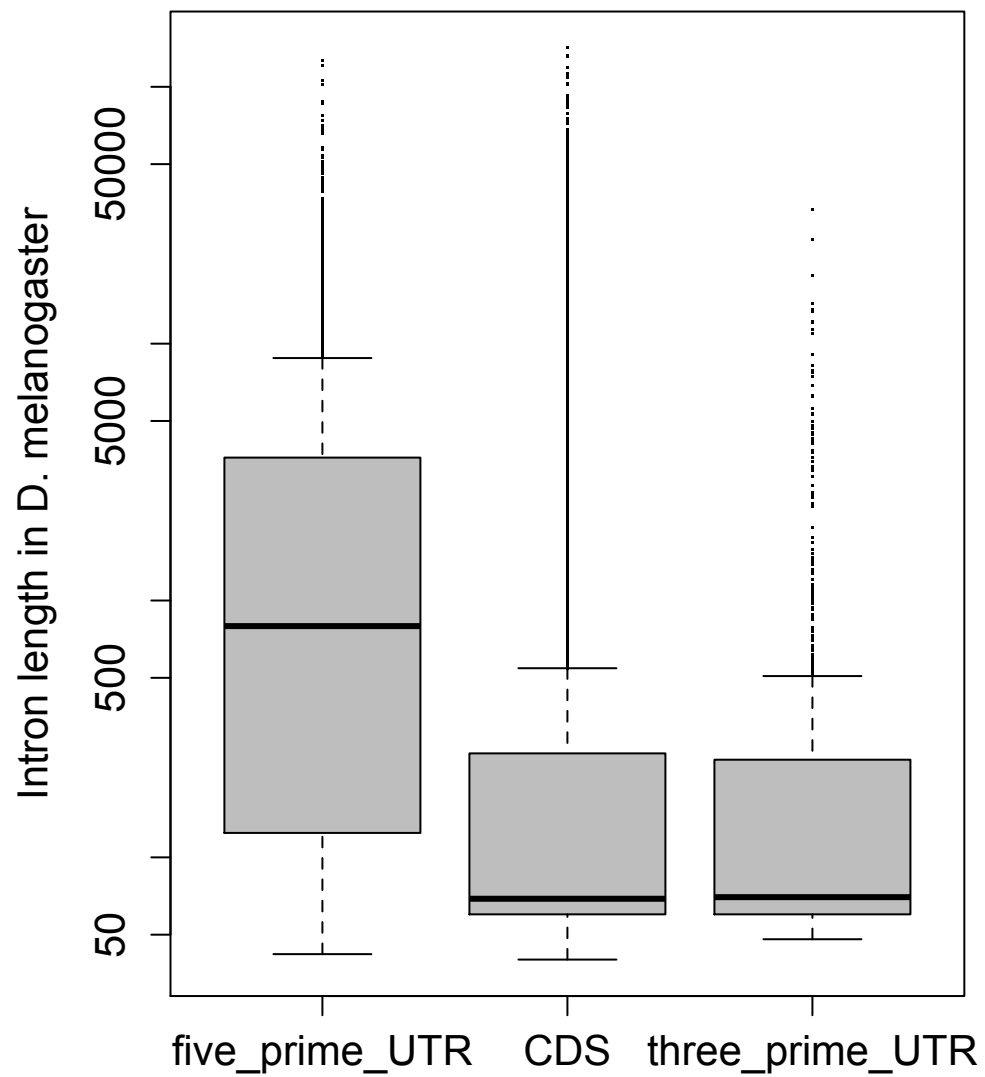

Supplement: Figure S8 — Intron length in different parts of the gene in D. pseudoobscura and D. melanogaster. Length comparison of introns located in 5′UTRs vs. CDS vs. 3′UTRs for D. pseudoobscura (a) and D. melanogaster (b). (PDF) [file pone.0046415.s008.pdf]
