# Supplementary material for: Evaluation of Different Reference Based Annotation Strategies Using RNA-Seq – A Case Study in Drososphila pseudoobscura
Source: PLoS One. 2012 Oct 3;7(10):e46415. doi: 10.1371/journal.pone.0046415 (PMC3463616; doi:10.1371/journal.pone.0046415)
Supplement: Table S1 — Mapping statistics. Proper pairs are defined as paired-end reads for which both mates are mapped to the reference genome. (DOC) [file pone.0046415.s009.doc]

### Table S1 – Mapping statistics

| **Strain** | **ps94m** | **ps94f** | **ps88m** | **ps88f** |
| --- | --- | --- | --- | --- |
| Total pairs | 42226987 | 42265943 | 41307715 | 42394057 |
| TopHat - Mapped | 34669533 | 37218321 | 31665277 | 37603857 |
| TopHat - Proper pairs | 23203318 | 22759125 | 19578778 | 24000489 |
| GSNAP - Mapped | 39603344 | 39998963 | 38438394 | 39990499 |
| GSNAP - Proper pairs | 36430057 | 38233958 | 35022273 | 38047262 |
