# Supplementary material for: Evaluation of Different Reference Based Annotation Strategies Using RNA-Seq – A Case Study in Drososphila pseudoobscura
Source: PLoS One. 2012 Oct 3;7(10):e46415. doi: 10.1371/journal.pone.0046415 (PMC3463616; doi:10.1371/journal.pone.0046415)
Supplement: Table S2 — Comparison of reference-based approaches for the sample ps94 females. Base level accuracy and percentage of confirmed junctions with different combinations of mapper and assembler on the sample ps94 females compared to the orthology annotation and the EST annotation (see Results). (DOC) [file pone.0046415.s010.doc]

### Table S2 – Comparison of reference based approaches for the sample ps94 females

| **Program combination** | ***Vs.* orthology annotation** | | ***Vs.* EST annotation** | |
| --- | --- | --- | --- | --- |
|  | **Base-level accuracy (%)** | **Confirmed junctions (%)** | **Base-level accuracy (%)** | **Confirmed junctions (%)** |
| TopHat + Cufflinks | 84.3 | 75.9 | 67.9 | 64.9 |
| GSNAP + Cufflinks | 81.4 | 74.4 | 69.9 | 63.7 |
| TopHat + Scripture | 72.9 | 67.7 | 64.0 | 64.7 |
