# Supplementary material for: Evaluation of Different Reference Based Annotation Strategies Using RNA-Seq – A Case Study in Drososphila pseudoobscura
Source: PLoS One. 2012 Oct 3;7(10):e46415. doi: 10.1371/journal.pone.0046415 (PMC3463616; doi:10.1371/journal.pone.0046415)
Supplement: Table S3 — Comparison of reference-based approaches for all the samples merged. Base level accuracy and percentage of confirmed junctions with different combinations of mapper and assembler for all the samples merged compared to the orthology annotation and the EST annotation (see Results) (DOC) [file pone.0046415.s011.doc]

### Table S3 – Comparison of reference based approaches for all the samples merged

| **Program combination** | ***Vs.* orthology annotation** | | ***Vs.* EST annotation** | |
| --- | --- | --- | --- | --- |
|  | **Base-level accuracy (%)** | **Confirmed junctions (%)** | **Base-level accuracy (%)** | **Confirmed junctions (%)** |
| TopHat + Cufflinks | 82.6 | 73.5 | 69.4 | 60.4 |
| GSNAP + Cufflinks | 47.4 | 42.5 | 41.7 | 35.1 |
| TopHat + Scripture | 39.2 | 35.4 | 34.0 | 32.4 |
