# Supplementary material for: Evaluation of Different Reference Based Annotation Strategies Using RNA-Seq – A Case Study in Drososphila pseudoobscura
Source: PLoS One. 2012 Oct 3;7(10):e46415. doi: 10.1371/journal.pone.0046415 (PMC3463616; doi:10.1371/journal.pone.0046415)
Supplement: Text S1 — Evaluation of Trinity on the FlyBase genes. (DOC) [file pone.0046415.s018.doc]

## Evaluation of Trinity on *D. pseudoobscura* FlyBase genes

We applied the de-novo transcriptome assembler Trinity to evaluate the reconstruction of the genes of the orthology annotation (see Material and Methods). To circumvent the memory intensive de novo assembly of the entire transcriptome, we implemented a gene-wise strategy and performed the isoform reconstruction on the sample ps94 males. For each gene of the orthology annotation we extracted the reads mapped by GSNAP overlapping the gene region using BEDTools and performed the reconstruction with Trinity (parameters --seqType fq --kmer_method meryl –paired fragment length 100). Then we mapped the resulting contig using BLAT (default parameters) against the *D. pseudoosbcura* r2.23 genome. For each transcript we extracted the best hit from BLAT and converted the output to GTF. Finally, we compared the obtained annotation to the orthology annotation with cuffcompare. We reached a base-level sensitivity of 47.9% and a junction-level sensitivity of 43.4%; both considerably lower than the accuracy of the reference-based assemblers (see Results).
